# Carbon Abatement and Emissions Associated with the Gasification of Walnut Shells for Bioenergy and Biochar Production

**DOI:** 10.1371/journal.pone.0150837

**Published:** 2016-03-10

**Authors:** Engil Isadora Pujol Pereira, Emma C. Suddick, Johan Six

**Affiliations:** Department of Plant Sciences, University of California Davis, One Shields Avenue, Davis, California, United States of America; Chinese Academy of Sciences, CHINA

## Abstract

By converting biomass residue to biochar, we could generate power cleanly and sequester carbon resulting in overall greenhouse gas emissions (GHG) savings when compared to typical fossil fuel usage and waste disposal. We estimated the carbon dioxide (CO_2_) abatements and emissions associated to the concurrent production of bioenergy and biochar through biomass gasification in an organic walnut farm and processing facility in California, USA. We accounted for (i) avoided-CO_2_ emissions from displaced grid electricity by bioenergy; (ii) CO_2_ emissions from farm machinery used for soil amendment of biochar; (iii) CO_2_ sequestered in the soil through stable biochar-C; and (iv) direct CO_2_ and nitrous oxide (N_2_O) emissions from soil. The objective of these assessments was to pinpoint where the largest C offsets can be expected in the bioenergy-biochar chain. We found that energy production from gasification resulted in 91.8% of total C offsets, followed by stable biochar-C (8.2% of total C sinks), offsetting a total of 107.7 kg CO_2_-C eq Mg^-1^ feedstock. At the field scale, we monitored gas fluxes from soils for 29 months (180 individual observations) following field management and precipitation events in addition to weekly measurements within three growing seasons and two tree dormancy periods. We compared four treatments: control, biochar, compost, and biochar combined with compost. Biochar alone or in combination with compost did not alter total N_2_O and CO_2_ emissions from soils, indicating that under the conditions of this study, biochar-prompted C offsets may not be expected from the mitigation of direct soil GHG emissions. However, this study revealed a case where a large environmental benefit was given by the waste-to-bioenergy treatment, addressing farm level challenges such as waste management, renewable energy generation, and C sequestration.

## Introduction

Converting farm residue into energy through gasification provides a solution for waste management, energy costs, and also curbs greenhouse gas (GHG) emissions derived from the usage of fossil fuels, but also challenges us to deal with the C-rich charcoal residue that remains after the thermo-transformation of the biomass. Since the early 2000’s, scientists have intensively investigated the use of this charcoal, known as biochar, as a soil amendment to improve various ecosystem services in agricultural lands. One of biochar’s claimed benefits is the decrease in soil nitrous oxide (N_2_O) emissions mainly related to N fertilization [1]. Because N_2_O has a global warming potential 296 times that of carbon dioxide (CO_2_), a suppression of its emissions can contribute to the total GHG mitigation potential of the bioenergy-biochar system [2]. Yet, increases in CO_2_ emissions from soils triggered by biochar application [3] and those associated with the mechanical incorporation of biochar in soils can compromise its GHG mitigation potential. Hence, an integrated assessment of the GHG fluxes associated to the bioenergy-biochar system will provide a thorough evaluation of the mitigation value of biochar.

The conversion of cellulosic waste biomass into energy can be achieved in a number of ways: through pyrolysis, gasification, torrefaction, and hydrothermal carbonization [4]. Each of these technologies differ in conditions, products, energy output, and char yield. Gasification is based on the reaction between biomass and limited amounts of oxygen and steam generating gaseous fuels (hydrogen and syngas) for power supply and C-rich biochar as residue [5]. Besides being a compelling alternative for the management of agricultural waste, the bioenergy-biochar system can contribute to the mitigation of climate change as the power generated by gasification replaces the use of energy from non renewable sources. Moreover, given that biochar-C is very stable and hardly decomposed by microorganisms, by incorporating biochar in the soil, substantial amounts of stable C remain buried in the soil for centuries to millennia [6].

Another potential co-benefit of biochar is the suppression of microbially-produced N_2_O gas. In soils, N_2_O is primarily produced as a by-product of nitrification and as an intermediate product of denitrification. Its rates of production are highly dependent on climatic conditions and soil properties including moisture, C availability, and supply and demand of N [7]. Indications of biochar decreasing N_2_O emissions were first observed in a laboratory incubation study performed by Yanai *et al*. [8] They found N_2_O fluxes decreased by 89% in biochar-amended soil when water-filled pore space (WFPS) was above 73%. They attributed these findings to potential water absorption by the biochar materials and improved aeration of the soil. Following Yanai *et al*. [8], several other studies investigated biochar effects on N_2_O fluxes, though outcomes were far from consistent and the underlying mechanisms remain unclear [9,10,11,12]. To better understand the N_2_O dynamics at the field-scale, it is important to consider that the N_2_O peaks are highly linked to how climate and farming management events affect the main drivers of N_2_O production, such as soil moisture, C and N availability [13]. Therefore, to fully exploit the potential of biochar to decrease N_2_O emissions, the monitoring of fluxes across an extensive array of agricultural and weather events is needed.

The increase of CO_2_ fluxes from the soil induced by biochar amendment can reduce the climate change mitigation potential of the bioenergy-biochar system. Soil fluxes of CO_2_ derive from the microbial decomposition of organic matter, plant material or root exudates, as well as, plant root respiration [14,15]. Although Karhu *et al*. [16] and Scheer *et al*. [17] have shown no influence of biochar on CO_2_ emissions, other studies suggest that biochar amendments increase CO_2_ fluxes from soils by altering microbial decomposition dynamics [18,19]. Novak *et al*. [20] in a laboratory incubation study, found that pecan-shell derived biochar induced the decomposition of co-amended switchgrass, resulting in significantly increased cumulative CO_2_ emissions. Zimmerman *et al*. [21], on the other hand, attributed increases in CO_2_ emissions from biochar-amended soils due to the mineralization of labile C from the biochar material. The monitoring of CO_2_ emissions upon biochar amendment is therefore crucial to determine potential tradeoffs between increases in soil CO_2_ emissions and GHG emissions savings from power and biochar production.

To evaluate the mitigation value of the bioenergy-biochar production system, this study capitalizes on an integrated walnut (*Juglans regia)* orchard and processing plant in the Central Valley of California, USA, which uses the walnut shell waste from the processing to produce energy to power its facilities. The objective of this study is to quantify the C offset provided by the gasification of walnut shells and amendment of the biochar in the soil, through the quantification of CO_2_ credits from energy generation and soil C sequestration, as well as direct GHG emissions from the soil. Furthermore, this study provides a long-term and high frequency assessment of the GHG fluxes from biochar amended-soils at the field-scale. We monitored CO_2_ and N_2_O fluxes over 29 months including 23 field operations and precipitation events and 180 individual observations in a 3.6 ha organic walnut orchard.

## Materials and Methods

### Structure of the study

To estimate C abatements and emissions associated with bioenergy-biochar production chain, we quantified:

1. the amount of avoided-CO_2_ emissions from displaced grid electricity by bioenergy (See subsection: Bioenergy and biochar production);
2. the CO_2_ emissions produced by farm machinery used during the field application of biochar (See subsection: Field trial establishment);
3. the amount of stable-C sequestered in the soil through biochar-C (See subsection: Field trial establishment);
4. the potential prompting and/or mitigation effect of biochar on direct CO_2_ and N_2_O emissions from soil (See subsection: Direct CO_2_ and N_2_O emissions from soils).

There were no emissions associated with land-use change as bioenergy and biochar resulted from the gasification of walnut shells residue. Additionally, the same facility hosted all the phases of this study from feedstock sourcing, to gasification, and to biochar incorporation, eliminating emissions associated with the transport of feedstock and biochar.

### Bioenergy and biochar production

This study was carried out on private land, in a commercial farm and processing facility in Winters, CA, USA(38° 31' 32” N, 121° 53' 57” W). The landowner, Mr. Russ Lester, authorized the study on this site. Dixon Ridge Farms (www.dixonridgefarms.com) is an organically-managed walnut (*Juglans regia*) orchard and processing facility that generates 910 Mg year^-1^ (1 Mega gram = 1 metric ton) of residue shells and converts it on-site into energy through gasification. Specifically, these walnut shells feed the downdraft gasifier (50 kW Biomax 50, Community Power Corporation, Littleton, CO, USA), which when supplemented with primary air (an influx of air to complete combustion) and heated at temperatures between 150°C and 500°C, decompose the shells into charcoal, gases, and tars. Then, the gases and charcoal were heated from 500°C to 900°C by the addition of small amounts of secondary air, which oxidizes the tars formed at lower temperatures. The resulting syngas is ignited in the cylinders of an internal combustion engine and the crankshaft spins an electrical generator. Under these conditions, for each 1.9 kg of shells, one kWh of net energy was produced along with biochar (2% of feedstock biomass). Electricity generated by the gasifier system is used primarily to power a large refrigeration system at Dixon Ridge Farms. To estimate the avoided CO_2_-C equivalent (CO_2_-C eq) emissions resulting from the generation of energy from gasification, we used the ‘Greenhouse Gas Equivalencies Calculator’ developed by the US-EPA; for every kWh the gasifier produced, 0.19 kg CO_2_-C eq were avoided [22] The resulting char was characterized by Mukome *et al* [23] and selected characteristics are presented in Table 1. Based on the life-cycle analyses of Roberts et al [24], we considered an 80% stability of biochar-C to estimate the amount of C biochar stores in the soil, which directly contributes to long-term C sequestration,

**Table 1:** Walnut shell biochar and soil (Yolo silt loam) physicochemical properties.

Biochar properties		Soil properties	
Production temperature (°C)	900	pH	7.5
Surface area (m^2^ g^-1^, BET)	227	Total C (g kg^-1^)	15.6
pH	9.7	Total N (g kg^-1^)	1.5
Ash content (%)	40.4	Potassium (mg kg^-1^)	312
Total carbon (%)	55.3	Olsen P (mg kg^-1^)	19.9
Organic carbon (%)	39.4	Sodium (mg kg^-1^)	47.6
Hydrogen:Carbon	0.22	Calcium (meq 100g^-1^)	11.3
PSD^a^ > 2 mm (%)	43.6	Magnesium (meq 100g^-1^)	14.4
1–2 mm (%)	19	Sand (%)	18.8
0.25–1 mm (%)	15	Silt (%)	47.6
< 0.25 mm (%)	22.4	Clay (%)	33.6

^a^SD = particle size distribution

### Field trial establishment

At the Dixon Ridge Farm, we established a randomized complete block design with three blocks and four treatments each: Control (no amendment), biochar (10 tons ha^-1^ or 47 kg N ha^-1^), compost (2 tons ha^-1^ or 47 kg N ha^-1^) and biochar + compost (5 tons of biochar ha^-1^ + 1 ton of compost ha^-1^, or 47 kg N ha^-1^). Compost treatments were included in the experiment to evaluate potential interactive effects between biochar and the main nutrient source in organic orchards. Additionally, each plot was divided into two functional locations: Tree row and tractor row. In the tractor row, measurements were taken in the center of the cover cropped area and represent a location of high N inputs due to the presence of cover crops. In the tree row, measurements were taken between trees and represent a location of high incidence of irrigation water and N uptake by the walnut trees. Each treatment plot comprised an area of 0.3 ha. The soil is a Yolo silt clay loam (Fine-silty, mixed, nonacid, thermic family of Mollic Xerofluvents) (USDA soil classification). Selected characteristic are presented in Table 1.

Biochar was applied to the experimental area using a compost sprayer and incorporated by three passes of a stubble-disk plow that opened the soil with a slicing action and distributed biochar vertically through a 15 cm depth. Three additional passes of a land leveler were required to allow subsequent cover crop mowing and harvest. A total of 12.2 hours per hectare of tractor (56 kW) use and 220 liters of diesel were required for all biochar incorporation procedures. For each liter of diesel consumed, 0.73 kg CO_2_-C eq or 2.6766 kg CO_2_ eq is produced [25]. We estimated GHG emissions from field machinery by multiplying the tractor weight by the amount of CO_2_-C eq emitted to produce 1 kg of machinery (3.49 kg CO_2_-C per kg of farm machinery) [26] correcting for the proportion of the farm machinery lifespan used for these activities [27]. We used a John Deere model 2755 (2,840 kg) for spreading and disking and a Caterpillar model D4D (4,535 kg) for land leveling.

**Fig 1:**
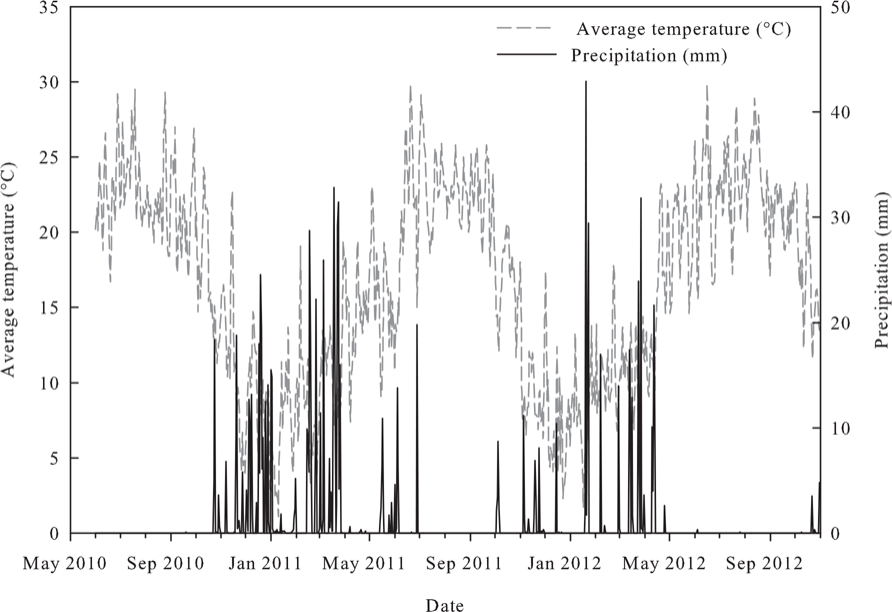
Temperature and precipitation data from May 2010 to October 2012 in Winters, CA, USA. Data is obtained from the California Irrigation Management Information System (CIMIS).

Gas and soil samplings were carried out for 29 months following the incorporation of the amendments on June 17^th^, 2010. The sampling periods are defined as growing season 1 (GS1; June to October 2010), tree dormancy 1 (TD1; October 2010 to May 2011), growing season 2 (GS2; June to October 2011), tree dormancy 2 (TD2; November to May 2012), and growing season 3 (GS3; June to October 2012). Local weather data (Fig 1) was obtained from the California Irrigation Management Information System (CIMIS). Total precipitation for GS1, TD1, GS2, TD2 and GS3 was 28, 584, 62, 333, and 9.2 mm, respectively. Average air temperatures for GS1, TD1, GS2, TD2 and GS3 were 21.3, 11.1, 21.3, 11.6 and 21.5°C, respectively.

It was estimated that leguminous cover crops contributed about 56 kg N ha^-1^ in the tree row and 103 kg N ha^-1^ in the tractor row. N supplied by the cover crop was calculated from biomass and %N content and then adjusted for the area of each functional location (i.e., tree and tractor rows) covered by the cover crop. Leguminous cover crops consisted of woollypod vetch (*Vicia dasycarpa*), purple vetch (*Vicia benghalensis* L.), common vetch (*Vicia sativa*), crimson clover (*Trifolium incarnatum* L.), sub-clover (*Trifolium subterraneum* L.), burr medic (*Medicago polymorpha* L.). During GS2, the entire experimental area received 123 kg N ha^-1^ as feather meal organic fertilizer (12% N) to sustain walnut production. Irrigation was applied as needed, using an overhead micro-sprinkler system.

### Direct CO_2_ and N_2_O emissions from soils

In situ soil-surface CO_2_ and N_2_O fluxes were sampled using a vented-closed-flux chamber method according to Hutchinson & Mosier [28]. The chambers were made out of polyvinylchloride (20.3 cm in diameter and 15 cm tall) with a rubber belt sealing between the top and bottom of the chamber. At sampling time, gas samples (20 mL) from each chamber were drawn from the headspace of the chambers with 25 mL polypropylene syringes and transferred into 12 mL pre-evacuated exetainers following fluxes of 20, 40 and 60 min after the chambers were installed. Samples were taken weekly or for seven days after an irrigation, precipitation, mowing, or fertilization event over the 29 months of experiment (For the list of events and date of occurrence see: S1 Table). N_2_O and CO_2_ concentrations were analyzed by electron capture (ECD) and thermal conductivity (TCD) gas chromatography, respectively (GC-2014 Shimadzu Gas Chromatograph, Kyoto, Japan). After calculation of the gas fluxes using linear and non-linear curve-fitting, the regression with the highest r^2^ values for each gas was expressed as a flux in either Mg CO_2_-C ha^-1^ day^-1^ or kg N_2_O–N ha^-1^ day^-1^. Cumulative CO_2_ and N_2_O emissions across the treatments and field locations were calculated by interpolation using trapezoidal integration for each time interval (between two gas samplings):

$$Cumulative\;gas\;emissions=\sum_{i}^{n}\frac{X_{i}+X_{i+1}}{2}(t_{i+1}-t_{i})$$

where *X*_*i*_ is the gas flux measurement on day *t*, *X*_i+1_ is the subsequent gas flux measured on day *t*_i+1_, and *n* is the final date of gas sampling.

### Ancillary measurements

Soil samples were collected three times within 7 days following each field operation (i.e., irrigation, fertilization, and cover-crop mowing) or precipitation event and once a week between events. At each soil sampling event, 2 replicate soil samples (0–15 cm depth) were taken from each plot within 1 m of each chamber using a 2 cm diameter auger and bulked for analyses. For quantification of soil ammonium (NH_4_^+^ -N) and nitrate (NO_3_^-^ -N), 15 g aliquots of soil were shaken for 1 h with 50 mL of 0.5 M K_2_SO_4_, and filtered through Whatman, No. 42, ashless filters. The concentration of NH_4_^+^-N and NO_3_^-^-N in the extracts were determined colorimetrically, by using the Berthelot reaction for NH_4_^+^-N and the vanadium (III) chloride reduction method for NO_3_^-^-N using a spectrophotometer (Shimadzu UV-PharmaSpec 1700, Nakagyo-ku, Japan) [29, 30].

Soil moisture content was determined gravimetrically by drying a subsample for 24 hours at 105°C. Water-filled pore space (WFPS) was calculated using measured bulk density and assuming a mineral particle size density of 2.65 g cm^-3^ [31]. Additionally, bulk soil samples were taken yearly from each plot and functional location by combining 2 soil cores (0–15 cm depth).

### Statistical analyses

To test the effects of the treatments, an ANOVA for the randomized block design was performed for the seasonal and event-based N_2_O and CO_2_ cumulative emissions. The analyses were performed using the mixed procedures in the SAS statistical package (SAS Institute, 2002) considering block as a random factor. Before subjecting the data to ANOVA, the homogeneity of variances and normality of the residuals were tested. If the requirements for ANOVA were not met, the data were log transformed. Pairwise comparisons were performed by the Tukey test. Significance was accepted at α < 0.05.

## Results

### CO_2_ abatements and emissions associated with gasification and biochar amendment

**Fig 2:**
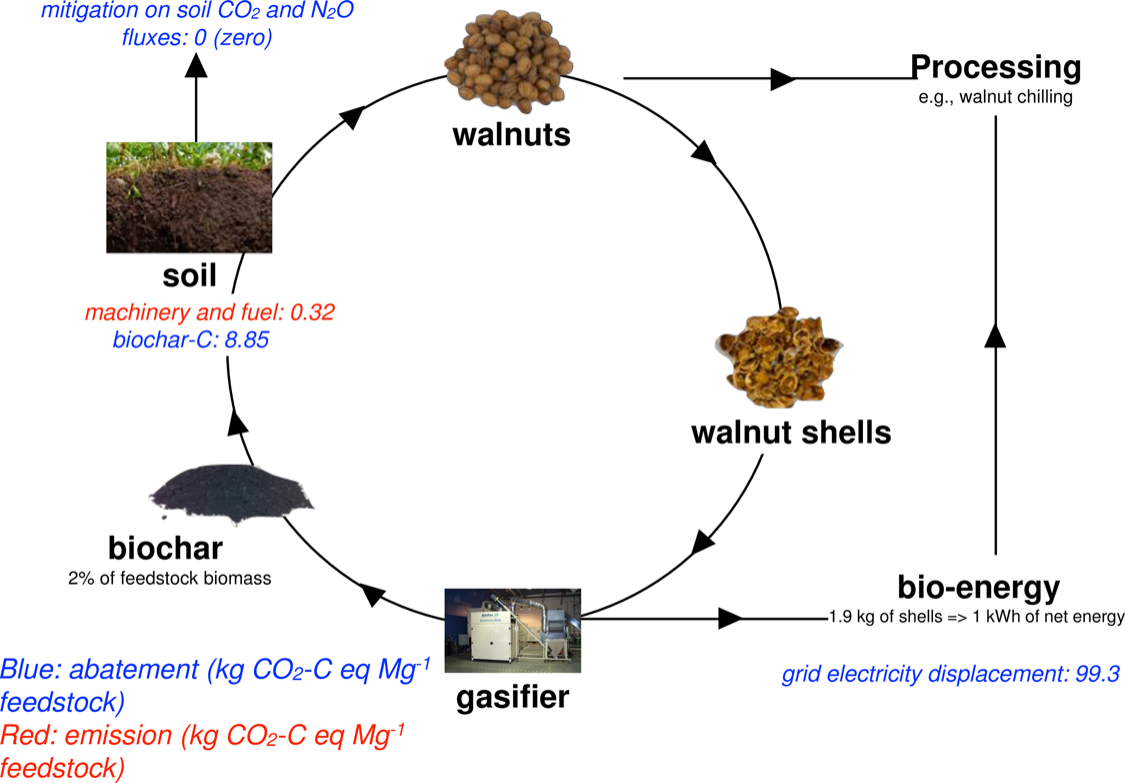
Illustration of the bioenergy-biochar production chain in a walnut farm and processing facility and the CO_2_ abatements and emissions associated to these activities.

Fig 2 shows the overall CO_2_ offsets and emissions produced by the conversion of walnut shells residue into energy and the incorporation of biochar in the soil. By gasifying 1 Mg (1 Mega gram = 1 metric ton) of walnut shells, this gasification unit generated 526 kWh of electricity and 20 kg of biochar as residue of gasification (2% feedstock mass) (Fig 2). Bioenergy production curbed 99.3 kg CO_2_-C Mg^-1^ feedstock or 0.19 kg CO_2_-C kWh^-1^ produced by not using electricity from the grid, representing the largest emission savings (91.8%) in the bioenergy-biochar production system. Biochar amendment buried 8.85 kg CO_2_-C Mg^-1^ feedstock in the soil, representing 8.2% of total C offsets. Machinery usage and fuel emissions from the field incorporation of biochar accounted for a total of 0.32 kg CO_2_-C Mg^-1^ feedstock. Accounting for the mitigation and emissions produced, overall, the bioenergy-biochar production system abated 107.7 kg CO_2_-C Mg^-1^ feedstock by converting the walnut shell residue into bioenergy and biochar.

### Direct CO_2_ and N_2_O emissions from soils

We did not observe any significant effect of biochar, compost or biochar + compost treatments on annual CO_2_ emissions (Table 2). Mean daily CO_2_ fluxes ranged from 0.0037 to 0.124 Mg CO_2_-C ha^-1^ day^-1^, and a high temporal and spatial variation was observed in all treatments (S1 and S2 Figs). Pronounced peaks of CO_2_ fluxes followed farming events and often times coincided with NO_3_^-^-N and NH_4_^+^-N peaks (S1 and S2 Figs).

**Table 2:** Cumulative CO_2_–C and N_2_O-N emissions by sampling year from both tree and tractor rows of a walnut orchard in Winters, CA, USA. Shown in parentheses is ± one standard error (n = 3). None of the treatments significantly altered the cumulative CO_2_–C and N_2_O-N emissions at *p* < 0.05.

Location	Treatment	CO_2_ emissions (Mg CO_2_-C ha^-1^)		N_2_O emissions (kg N_2_O-N ha^-1^)	
		Year 1	Year 2	Year 1	Year 2
Tree row	Control	6.64 (0.63)	6.75 (0.13)	1.15 (0.24)	1.18 (0.02)
	Biochar	5.85 (0.60)	6.32 (0.50)	0.94 (0.19)	1.33 (0.18)
	Compost	5.60 (0.30)	6.79 (0.90)	0.97 (0.11)	0.97 (0.07)
	Biochar+compost	5.15 (0.05)	6.44 (0.61)	1.03 (0.17)	1.25 (0.18)
	*p-value*	*0*.*21*	*0*.*72*	*0*.*91*	*0*.*25*
Tractor row	Control	5.55 (0.55)	9.33 (0.61)	1.29 (0.20)	2.41 (0.37)
	Biochar	5.54 (0.18)	8.19 (0.42)	1.02 (0.09)	2.09 (0.30)
	Compost	6.18 (0.47)	9.68 (0.73)	1.34 (0.06)	3.09 (0.57)
	Biochar+compost	5.59 (0.49)	10.1 (0.41)	1.32 (0.14)	2.93 (0.61)
	*p-value*	*0*.*93*	*0*.*17*	*0*.*29*	*0*.*46*

Year 1 = period between June 2010 to May 2011; Year 2 = period between June 2011 to May 2012.

Analyses of annual cumulative N_2_O emissions indicated no treatment effect at the significance level of p < 0.05 (Table 2). Additionally, results of ancillary measurements also indicated no effects of biochar on important factors controlling N_2_O emissions (WFPS, NH_4_^+^-N and NO_3_^-^-N) (S1 and S2 Figs). Overall mean cumulative N_2_O emissions were 1.52 kg N_2_O-N ha^-1^ year^-1^, equivalent to 0.19 Mg CO_2_-Ce ha^-1^ year^-1^. During the growing seasons, mean cumulative N_2_O emissions ranged from 0.46 to 2.44 kg N_2_O-N ha^-1^ season^-1^, while during tree dormancy fluxes ranged from 0.18 to 0.69 kg N_2_O-N ha^-1^ season^-1^. Overall mean daily N_2_O fluxes ranged from 4.8 x 10^−4^ to 9.7 x 10^−2^ kg N_2_O-N ha^-1^ day^-1^, and a high temporal and spatial variation was observed for all treatments. Peaks of N_2_O fluxes followed precipitation and farming management events, which often coincided with WFPS, NO_3_^-^-N, and NH_4_^+^-N peaks (S1 and S2 Figs). Events involving resource inputs such as fertilization or cover crop mowing induced the largest N_2_O peaks with average 0.30 kg N_2_O-N ha^-1^ event^-1^.

We evaluated potential treatment effects on the cumulative CO_2_ and N_2_O emissions following major field operation or precipitation events that occurred at the orchard (S4–S13 Tables). For most of the events, CO_2_ emissions were not altered by the amendments, except following an irrigation event in GS3 where biochar in combination with compost increased 41% of CO_2_ emissions in the tractor row. Similarly, N_2_O emissions was not affected by the amendments during most of field operation or precipitation events, except during an irrigation event during GS2 where biochar decreased about 71% N_2_O emissions of in the tractor row.

**Fig 3:**
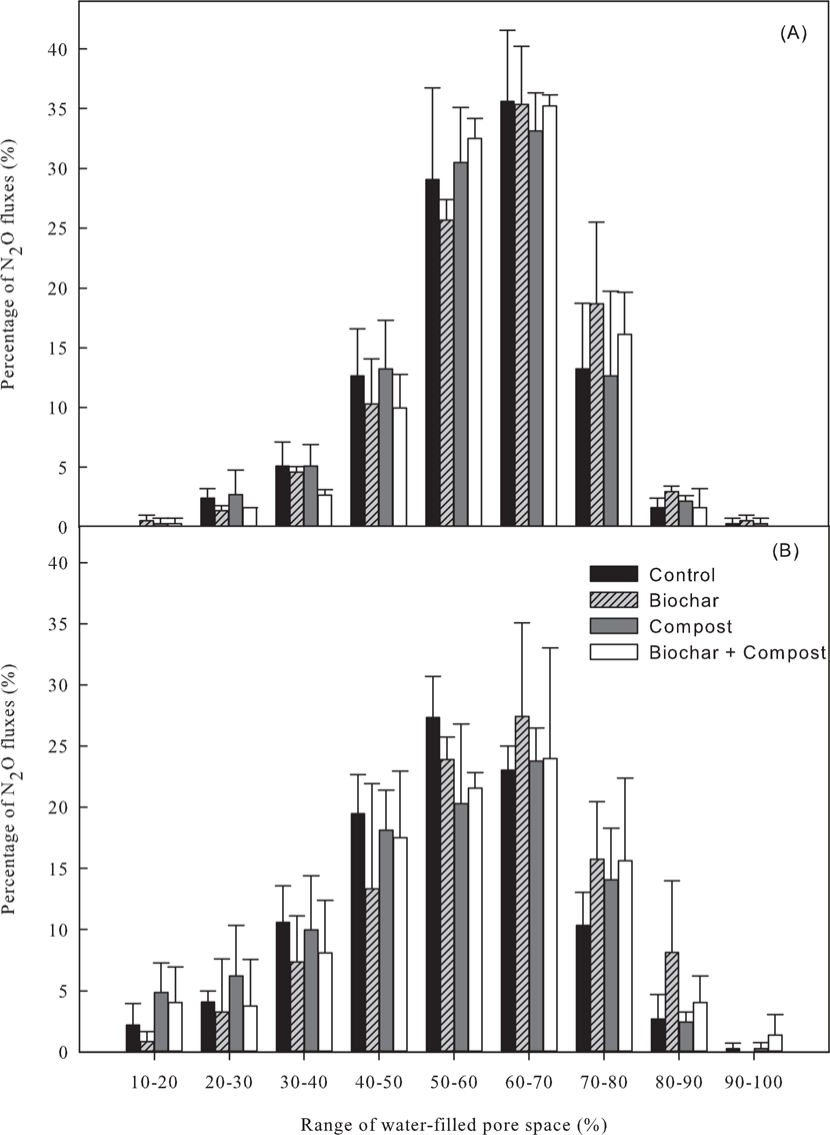
Distribution of sampling gas fluxes across the different ranges of water-filled pore space during 29 months of observations (June 2010 to October 2012). Error bars indicate standard deviation (n = 3).

Moisture data indicated that about 52.2% of all sampling gas fluxes occurred at < 60% WFPS; 29.7% of fluxes occurred between 60 and 70% WFPS; and 18.1% of fluxes occurred > 70% WFPS, with no differences between the treatments (Fig 3).

## Discussion

### Mitigating direct CO_2_ and N_2_O emissions from soils

We did not detect any significant effects of the treatments on annual or seasonal cumulative CO_2_ emissions. Increases in CO_2_ emissions from biochar-amended soils are presumably due to the mineralization of residual labile C present in the biochar or due to a priming of native soil organic matter decomposition [3, 21, 32]. Bruun *et al*. [32] suggested that biochar mineralization decreases with increases in its production temperature. In our study we examined the effect of walnut shell biochar produced at 900°C, a considerably higher temperature than the typical temperature of 350–650°C at which biochar is usually produced according to the literature [19, 33]. An incubation study performed by Mukome *et al*. [34] also reported no changes in CO_2_ fluxes from soils amended with the same walnut shell biochar, compared to non-amended soil. They also reveled that the walnut shell biochar has a very low H/C ratio (H/C = 0.22), which indicates a high level of aromaticity and therefore, stability in the soil [35, 36]. Thus, high temperature gasification produced a walnut shell biochar very recalcitrant, stable and lacking labile C for decomposition to CO_2_.

The analysis of the N_2_O fluxes sampled during 29 months indicated that biochar alone or in combination with compost did not alter cumulative N_2_O emissions. The lack of effects of biochar on N_2_O emissions is in agreement with other field trials in California. The same walnut shell biochar did not alter N_2_O emissions during 14 months in a small scale vegetable cropping system [37] and during 24 months in a commercial wine grape system [38]. Suggested mechanisms by which biochar could decrease N_2_O fluxes include: (i) biochar’s highly porous structure decreases anaerobic sites for N_2_O production [8], (ii) biochar’s alkalinity increases soil pH, favoring N_2_ over N_2_O production [39]; and (iii) microbial immobilization of NO_3_^-^-N [40, 41]. These mechanisms directly disturb N_2_O produced from denitrification, which may not be the predominant source of N_2_O at our site due to scarce precipitation (Fig 1). Although this study did not distinguish N_2_O sources, it is well known that nitrification produces most N_2_O in soils at 35–60% WFPS; whereas, above 70% WFPS, denitrification predominates as the source of N_2_O fluxes [42]. With 52.2% of the fluxes occurring under 60% WFPS and only 18.1% of the fluxes occurring higher than 70% WFPS (Fig 3), potential decreases in denitrification derived-N_2_O are most likely not reflected in the overall N_2_O emissions. In an incubation study, van Zwieten *et al*. [11] quantified N_2_O fluxes from soils amended with five biochar types at 65–70% WFPS and at 100% WFPS. They observed that, in all cases, biochar materials decreased N_2_O fluxes only when WFPS was near 100%, and no effects were observed at 65–70% WFPS. Therefore, we suggest that the effects of biochar on N_2_O emissions may not be observed in sites located under modest precipitation and irrigation regimes, such as under the Mediterranean climate of California. Further studies should be performed to confirm the association of biochar effects with the denitrification process.

Of the 23 farm management and precipitation events, isolated decreases of N_2_O emissions by biochar amendment were observed following a precipitation event and an irrigation event. In both cases, ancillary measurements could not explain the observed effects, as there were no changes in WFPS, NO_3_^-^-N nor NH_4_^+^-N due to biochar during those events. Furthermore, these decreases were not sufficiently large to be reflected in their respective seasonal cumulative N_2_O emissions. This highlights the importance of long-term and high frequency sampling to prevent short-term trends from being extrapolated into total seasonal emissions.

While this study in particular does not show significant influences on greenhouse gas emissions, water holding capacity, or soil mineral N concentrations, walnut shell biochar has been shown in other conditions to foster soil-microbial interactions augmenting nutrient cycling in organic systems [43]. With the California walnut industry valued around 1.8 billion USD and generating 99% of U.S. walnut production (600,000 tons per year) [44] combined with the growing adoption of gasification units by agro-industries interested in bioenergy, make the research opportunities and knowledge gaps in determining the benefits of walnut shell biochar increasingly relevant, especially in the areas of soil nutrient dynamics and crop production efficiency.

### CO_2_ offsets from the bioenergy-biochar production system

Biomass gasification attracts many agricultural enterprises interested in achieving energy self-sufficiency, while managing the disposal of large amounts of production waste. Claimed improvements in soil properties, ecosystems services (i.e., yield increases, C sequestration, decreases in soil GHG emissions), and a perspective of product marketing also make the residue from gasification, biochar, a valuable product. Among the estimated sources for C abatement, energy production from gasification provided the greatest contribution, accounting for 91.8% of the total C offsets, followed by the C stable in the soil as biochar (8.2% of the total C offsets). Electricity produced from gasification provides a greater contribution to total C offsets in the bioenergy-biochar production chain than pyrolysis units. Hammond *et al*. [45] investigated a variety of feedstocks and found that the contribution of electricity to overall C abatement ranges between 10–25%. On the other hand, pyrolysis units produce biochars with higher C abatement potential than gasification units, ranging from 40 to 66% [24, 45]. Furthermore, the CO_2_-C abatements created by pyrolysis systems have been reported to be over 2-fold larger than the obtained with gasification in this study (107.7 CO_2_-C Mg^-1^ feedstock) [24], likely due to the higher yield of biochar-C from pyrolysis of biomass. The feedstock type and thermo-transformation conditions determine the proportion of each output. When targeting energy output, units are designed to maximize the energy extraction from the biomass at very high temperatures and the resulting biochar-C is highly recalcitrant. [46, 47] When targeting biochar-C, units tend to operate at lower temperatures (< 600°C), yielding high amounts of charcoal that contain a larger labile C fraction compared to biochar produced at higher temperatures. Because labile C is rapidly decomposed in the soil, it is important to consider the amount of stable C in order not to overestimate the CO_2_ abatements offered by the system [48].

Among the estimated sources of C emissions, biochar amendment operations led to a slight reduction in the overall C abatements. It is important to notice; however, that the same facility hosted all the phases of this study, there were no emissions associated with the transport of feedstock and biochar. Depending on the distance, emissions from road transport of feedstock to gasification facility and biochar to fields can substantially offset the GHG benefits of gasification.

## Conclusion

This study investigated the climate change mitigation potential of the bioenergy-biochar production system by estimating the C abatement and emissions associated with the gasification of walnut shells for power production and soil amendment of biochar. We collaborated with a commercial walnut farm and processing facility that hosted the all the stages of this study from walnut cultivation, to processing, to gasification, and to biochar incorporation. Under the conditions of this study, the bioenergy-biochar production system appears as a valuable technology for creating C credits by replacing the use of non-renewable energy and burying stable C in the soil. As an approach to decrease direct GHG emissions from soils, however, the amendment of biochar did not reduce N_2_O emissions over the 29 months of high frequency observations at the field scale. We also showed that biochar did not alter annual or seasonal CO_2_ fluxes from soil, possibly due to its stable and recalcitrant matrix, which is beneficial for the purposes of soil C sequestration. In conclusion, our results indicate that biochar prompted C abatements only from the gasification of biomass residue and C burial with no effects on direct CO_2_ or N_2_O emissions from the soil.

## Supporting Information

S1 Fig **Comparison of biochar and compost amendments in the tree row in (a) CO**_**2**_ **emissions, (b) N**_**2**_**O emissions, (c) WFPS, (d) NO**_**3**_^-^**-N, and (e) NH**_**4**_^**+**^**-N from measurements taken during the 29 months of observations (June 2010 to October 2012).** Error bars represent ± one standard error (n = 3).
(PDF)

S2 Fig **Comparison of biochar and compost amendments in the tractor row in (a) CO_2_ emissions, (b) N_2_O emissions, (c) WFPS, (d) NO_3_^-^-N, and (e) NH_4_^+^-N from measurements taken during the 29 months of observations (June 2010 to October 2012).** Error bars represent ± one standard error (n = 3).
(PDF)

S1 Table List of management and precipitation events that occurred at Dixon Ridge Farm (Winters, CA, USA) during the 29 months of this study. (PDF)

S2 Table Cumulative CO_2_–C emissions by sampling season from both tree and tractor rows of a walnut orchard in Winters, CA, USA. Shown in parentheses is ± one standard error (n = 3). None of the treatments significantly altered the cumulative CO_2_–C emissions at *p* < 0.05.
(PDF)

S3 Table Cumulative N_2_O-N emissions by sampling season from both tree and tractor rows of a walnut orchard in Winters, CA, USA. Shown in parentheses is ± one standard error (n = 3). None of the treatments significantly altered the cumulative N_2_O-N emissions at *p* < 0.05.
(PDF)

S4 Table Cumulative CO_2_ emissions by event that occurred during growing season 1 (GS1), period between June and October 2010, from both tree and tractor rows of a walnut orchard in Winters, CA, USA. Shown in parentheses is ± one standard error (n = 3). None of the treatments significantly altered the cumulative CO_2_ emissions at *p* < 0.05.
(PDF)

S5 Table Cumulative CO_2_ emissions by event that occurred during tree dormancy 1 (TD1), period between November 2010 and May 2011, from both tree and tractor rows of a walnut orchard in Winters, CA, USA. Shown in parentheses is ± one standard error (n = 3). None of the treatments significantly altered the cumulative CO_2_ emissions at *p* < 0.05.
(PDF)

S6 Table Cumulative CO_2_ emissions by event that occurred during growing season 2 (GS2), period between June and October 2011, from both tree and tractor rows of a walnut orchard in Winters, CA, USA. Shown in parentheses is ± one standard error (n = 3). None of the treatments significantly altered the cumulative CO_2_ emissions at *p* < 0.05.
(PDF)

S7 Table Cumulative CO_2_ emissions by event that occurred during tree dormancy 2 (TD2), period between November 2011 and May 2012, from both tree and tractor rows of a walnut orchard in Winters, CA, USA. Shown in parentheses is ± one standard error (n = 3). None of the treatments significantly altered the cumulative CO_2_ emissions at *p* < 0.05.
(PDF)

S8 Table Cumulative CO_2_ emissions by event that occurred during growing season 3 (GS3), period between June and October 2012, from both tree and tractor rows of a walnut orchard in Winters, CA, USA. Shown in parentheses is ± one standard error (n = 3). Means followed by different letter within a column are statistically different at *p* > 0.05.
(PDF)

S9 Table Cumulative N_2_O emissions by event that occurred during growing season 1 (GS1), period between June and October 2010, from both tree and tractor rows of a walnut orchard in Winters, CA, USA. Shown in parentheses is ± one standard error (n = 3). None of the treatments significantly altered the cumulative N_2_O emissions at *p* < 0.05.
(PDF)

S10 Table Cumulative N_2_O emissions by event that occurred during tree dormancy 1 (TD1), period between November 2010 and May 2011, from both tree and tractor rows of a walnut orchard in Winters, CA, USA. Shown in parentheses is ± one standard error (n = 3). Means followed by different letter within a column are statistically different at *p* > 0.05.
(PDF)

S11 Table Cumulative N_2_O emissions by event that occurred during growing season 2 (GS2), period between June and October 2011, from both tree and tractor rows of a walnut orchard in Winters, CA, USA. Shown in parentheses is ± one standard error (n = 3). Means followed by different letter within a column are statistically different at *p* > 0.05.
(PDF)

S12 Table Cumulative N_2_O emissions by event that occurred during tree dormancy 1 (TD1), period between November 2010 and May 2011, from both tree and tractor rows of a walnut orchard in Winters, CA, USA. Shown in parentheses is ± one standard error (n = 3). None of the treatments significantly altered the cumulative N_2_O emissions at *p* < 0.05.
(PDF)

S13 Table Cumulative N_2_O emissions by event that occurred during growing season 1 (GS1), period between June and October 2010, from both tree and tractor rows of a walnut orchard in Winters, CA, USA. Shown in parentheses is ± one standard error (n = 3). None of the treatments significantly altered the cumulative N_2_O emissions at *p* < 0.05.
(PDF)

## References

[1] Cayuela M, Van Zwieten L, Singh B, Jeffery S, Roig A, Sánchez-Monedero M. Biochar's role in mitigating soil nitrous oxide emissions: A review and meta-analysis. Agriculture, Ecosystems & Environment. 2014;191:5–16.

[2] Gaunt J, Cowie A. Biochar, greenhouse gas accounting and emissions trading. Biochar for environmental management: Science and technology. 2009:317–40.

[3] Wardle DA, Nilsson M-C, Zackrisson O. Fire-derived charcoal causes loss of forest humus. Science. 2008;320(5876):629–. 10.1126/science.1154960 18451294

[4] Spokas KA, Cantrell KB, Novak JM, Archer DW, Ippolito JA, Collins HP, et al Biochar: a synthesis of its agronomic impact beyond carbon sequestration. Journal of Environmental Quality. 2012;41(4):973–89. 10.2134/jeq2011.0069 22751040

[5] Digman B, Joo HS, Kim DS. Recent progress in gasification/pyrolysis technologies for biomass conversion to energy. Environmental progress & sustainable energy. 2009;28(1):47–51.

[6] Cheng CH, Lehmann J, Thies JE, Burton SD. Stability of black carbon in soils across a climatic gradient. Journal of Geophysical Research: Biogeosciences (2005–2012). 2008;113(G2).

[7] Firestone MK, Davidson EA, Andreae M, Schimel D. Microbiological basis of NO and N2O production and consumption in soil. Exchange of trace gases between terrestrial ecosystems and the atmosphere. 1989:7–21.

[8] Yanai Y, Toyota K, Okazaki M. Effects of charcoal addition on N2O emissions from soil resulting from rewetting air-dried soil in short-term laboratory experiments. Soil Science and Plant Nutrition. 2007;53(2):181–8.

[9] Spokas KA, Reicosky DC. Impacts of sixteen different biochars on soil greenhouse gas production. Annals of Environmental Science. 2009;3(1):4.

[10] Singh BP, Hatton BJ, Singh B, Cowie AL, Kathuria A. Influence of biochars on nitrous oxide emission and nitrogen leaching from two contrasting soils. Journal of environmental quality. 2010;39(4):1224–35. 20830910 10.2134/jeq2009.0138

[11] Van Zwieten L, Kimber S, Morris S, Downie A, Berger E, Rust J, et al Influence of biochars on flux of N2O and CO2 from Ferrosol. Soil Research. 2010;48(7):555–68.

[12] Cayuela ML, Sánchez-Monedero MA, Roig A, Hanley K, Enders A, Lehmann J. Biochar and denitrification in soils: when, how much and why does biochar reduce N2O emissions? Scientific reports. 2013;3. 10.1038/srep01732 PMC3635057 23615819

[13] Smith KA, Dobbie KE. The impact of sampling frequency and sampling times on chamber‐based measurements of N2O emissions from fertilized soils. Global Change Biology. 2001;7(8):933–45.

[14] Hanson P, Edwards N, Garten C, Andrews J. Separating root and soil microbial contributions to soil respiration: a review of methods and observations. Biogeochemistry. 2000;48(1):115–46.

[15] Yiqi L, Zhou X. Soil respiration and the environment: Academic press; 2010.

[16] Karhu K, Mattila T, Bergström I, Regina K. Biochar addition to agricultural soil increased CH4 uptake and water holding capacity–Results from a short-term pilot field study. Agriculture, Ecosystems & Environment. 2011;140(1):309–13.

[17] Scheer C, Grace PR, Rowlings DW, Kimber S, Van Zwieten L. Effect of biochar amendment on the soil-atmosphere exchange of greenhouse gases from an intensive subtropical pasture in northern New South Wales, Australia. Plant and Soil. 2011;345(1–2):47–58.

[18] Kuzyakov Y, Subbotina I, Chen H, Bogomolova I, Xu X. Black carbon decomposition and incorporation into soil microbial biomass estimated by 14C labeling. Soil Biology and Biochemistry. 2009;41(2):210–9.

[19] Luo Y, Durenkamp M, De Nobili M, Lin Q, Brookes P. Short term soil priming effects and the mineralisation of biochar following its incorporation to soils of different pH. Soil Biology and Biochemistry. 2011;43(11):2304–14.

[20] Novak JM, Busscher WJ, Watts DW, Laird DA, Ahmedna MA, Niandou MA. Short-term CO2 mineralization after additions of biochar and switchgrass to a typic Kandiudult. Geoderma. 2010;154(3):281–8.

[21] Zimmerman AR, Gao B, Ahn M-Y. Positive and negative carbon mineralization priming effects among a variety of biochar-amended soils. Soil Biology and Biochemistry. 2011;43(6):1169–79.

[22] Environmental Protection Agency (EPA- USA). eGRID, U.S. annual non-baseload CO_2_ output emission rate, year 2014 data, U.S. Environmental Protection Agency, Washington, DC. 2014. Available: http://www.epa.gov/cleanenergy/energy-resources/refs.html

[23] Mukome FN, Zhang X, Silva LC, Six J, Parikh SJ. Use of chemical and physical characteristics to investigate trends in biochar feedstocks. Journal of agricultural and food chemistry. 2013;61(9):2196–204. 10.1021/jf3049142 23343098 PMC4154706

[24] Roberts KG, Gloy BA, Joseph S, Scott NR, Lehmann J. Life cycle assessment of biochar systems: Estimating the energetic, economic, and climate change potential. Environmental science & technology. 2009;44(2):827–33. 10.1021/es902266r 20030368

[25] Department for Environment, Food & Rural Affairs (Defra–UK). Guidelines for Company Reporting on Greenhouse Gas Emissions. Annexes updated July 2005. Annex 1 –Fuel Conversion Factors. Available: http://www.defra.gov.uk/environment/business/envrp/gas/envrpgas-annexes.pdf

[26] Wells, C. Total Energy Indicators of Agricultural Sustainability: Diary farming study. Technical paper 2001/3 Ministry of Agriculture and Forestry, New Zealand. 2001. Available: http://maxa.maf.govt.nz/mafnet/publications/techpapers/techpaper0103-dairy-farming-case-study.pdf

[27] Maraseni TN, Mushtaq S, Hafeez M, Maroulis J. Greenhouse gas implications of water reuse in the Upper Pumpanga River integrated irrigation system, Philippines. Agricultural water management. 2010;97(3):382–8.

[28] Hutchinson G, Mosier A. Improved soil cover method for field measurement of nitrous oxide fluxes. Soil Science Society of America Journal. 1981;45(2):311–6.

[29] Alef K, Nannipieri P. Methods in applied soil microbiology and biochemistry: Academic press; 1995.

[30] Doane TA, Horwáth WR. Spectrophotometric determination of nitrate with a single reagent. Analytical Letters. 2003;36(12):2713–22.

[31] Robertson G, Groffman P. Nitrogen transformations. Soil microbiology, ecology, and biochemistry. 2007:341–64.

[32] Bruun S, Jensen ES, Jensen LS. Microbial mineralization and assimilation of black carbon: Dependency on degree of thermal alteration. Organic Geochemistry. 2008;39(7):839–45.

[33] Biederman LA, Harpole WS. Biochar and its effects on plant productivity and nutrient cycling: a meta‐analysis. GCB Bioenergy. 2013;5(2):202–14.

[34] Hammes K, Smernik RJ, Skjemstad JO, Schmidt MW. Characterisation and evaluation of reference materials for black carbon analysis using elemental composition, colour, BET surface area and 13 C NMR spectroscopy. Applied Geochemistry. 2008;23(8):2113–22.

[35] Mukome FN, Six J, Parikh SJ. The effects of walnut shell and wood feedstock biochar amendments on greenhouse gas emissions from a fertile soil. Geoderma. 2013;200:90–8.

[36] Manya JJ, Velo E, Puigjaner L. Kinetics of biomass pyrolysis: a reformulated three-parallel-reactions model. Industrial & engineering chemistry research. 2003;42(3):434–41.

[37] Suddick EC, Six J. An estimation of annual nitrous oxide emissions and soil quality following the amendment of high temperature walnut shell biochar and compost to a small scale vegetable crop rotation. Science of the Total Environment. 2013;465:298–307. 10.1016/j.scitotenv.2013.01.094 23490323

[38] Verhoeven E, Six J. Biochar does not mitigate field-scale N_2_O emissions in a Northern California vineyard: An assessment across two years. Agriculture, Ecosystems & Environment. 2014;191:27–38.

[39] Zheng J, Stewart CE, Cotrufo MF. Biochar and nitrogen fertilizer alters soil nitrogen dynamics and greenhouse gas fluxes from two temperate soils. Journal of environmental quality. 2012;41(5):1361–70. 10.2134/jeq2012.0019 23099927

[40] Felber R, Leifeld J, Horák J, Neftel A. Nitrous oxide emission reduction with greenwaste biochar: comparison of laboratory and field experiments. European Journal of Soil Science. 2014;65(1):128–38.

[41] Case SD, McNamara NP, Reay DS, Whitaker J. The effect of biochar addition on N2O and CO2 emissions from a sandy loam soil–The role of soil aeration. Soil Biology and Biochemistry. 2012;51:125–34.

[42] Bateman E, Baggs E. Contributions of nitrification and denitrification to N2O emissions from soils at different water-filled pore space. Biology and Fertility of Soils. 2005;41(6):379–88.

[43] Pereira EIP, Suddick EC, Mukome FN, Parikh SJ, Scow K, Six J. Biochar alters nitrogen transformations but has minimal effects on nitrous oxide emissions in an organically managed lettuce mesocosm. Biology and Fertility of Soils. 2015:1–10.

[44] California Department of Food and Agriculture. California County Agricultural Commissioners’ Reports. 2012. Available: http://www.nass.usda.gov/Statistics_by_State/California/Publications/AgComm/201212cactb00.pdf

[45] Hammond J, Shackley S, Sohi S, Brownsort P. Prospective life cycle carbon abatement for pyrolysis biochar systems in the UK. Energy Policy. 2011;39(5):2646–55.

[46] Ahmed I, Gupta A. Syngas yield during pyrolysis and steam gasification of paper. Applied energy. 2009;86(9):1813–21.

[47] Brewer CE, Schmidt‐Rohr K, Satrio JA, Brown RC. Characterization of biochar from fast pyrolysis and gasification systems. Environmental Progress & Sustainable Energy. 2009;28(3):386–96.

[48] Bruun EW, Hauggaard-Nielsen H, Ibrahim N, Egsgaard H, Ambus P, Jensen PA, et al Influence of fast pyrolysis temperature on biochar labile fraction and short-term carbon loss in a loamy soil. Biomass and Bioenergy. 2011;35(3):1182–9.

