# Supplementary material for: Carbon Abatement and Emissions Associated with the Gasification of Walnut Shells for Bioenergy and Biochar Production
Source: PLoS One. 2016 Mar 10;11(3):e0150837. doi: 10.1371/journal.pone.0150837 (PMC4786142; doi:10.1371/journal.pone.0150837)

S2 Fig: Comparison of biochar and compost amendments in the tractor row in (a) CO_2 emissions, (b) N_2O emissions, (c) WFPS, (d) NO_3^--N, and (e) NH_4^+-N from measurements taken during the 29 months of observations (June 2010 to October 2012). Error bars represent ± one standard error (n = 3).

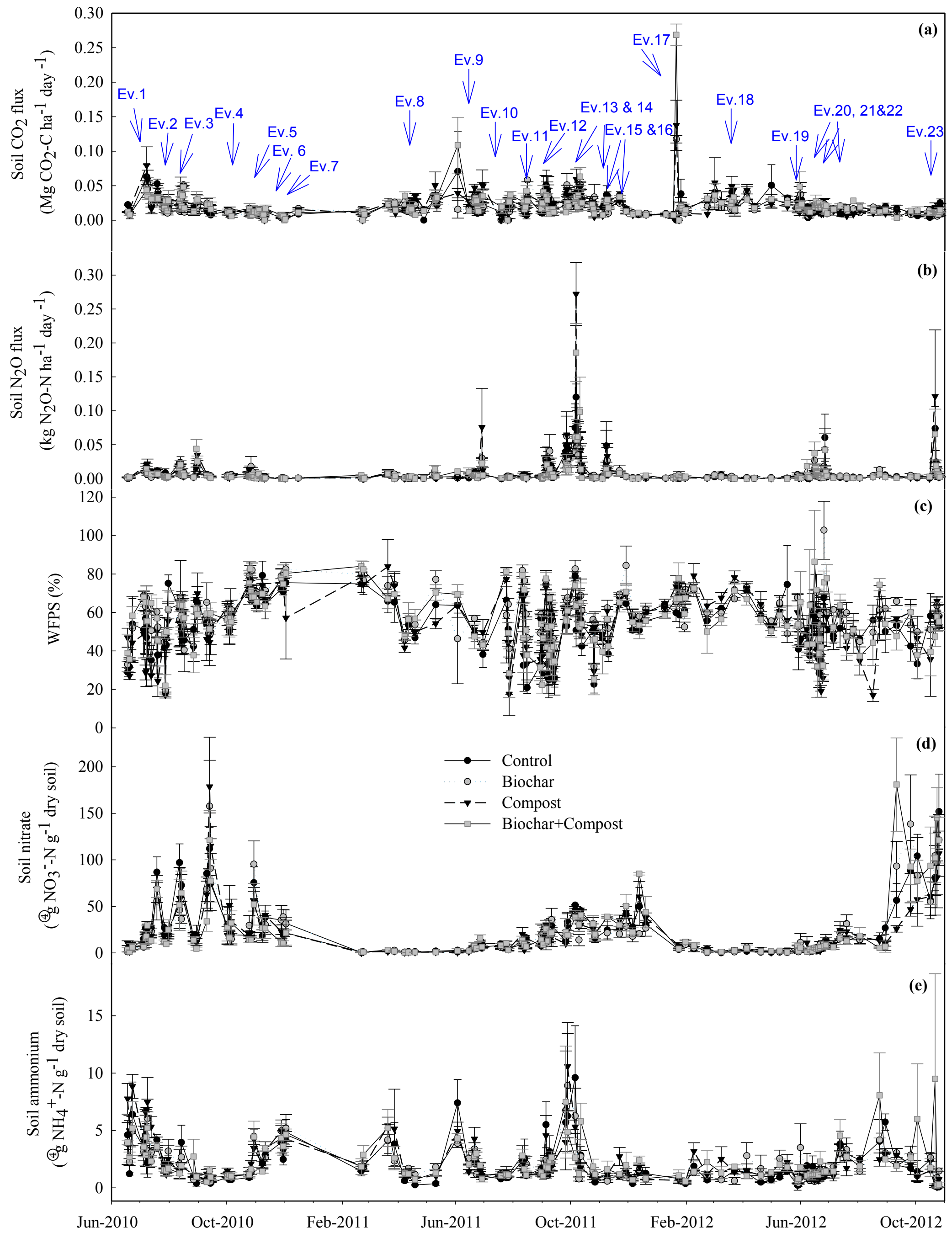

Supplement: S2 Fig — Comparison of biochar and compost amendments in the tractor row in (a) CO2 emissions, (b) N2O emissions, (c) WFPS, (d) NO3--N, and (e) NH4+-N from measurements taken during the 29 months of observations (June 2010 to October 2012). Error bars represent ± one standard error (n = 3). (PDF) [file pone.0150837.s002.pdf]
