# Supplementary material for: Carbon Abatement and Emissions Associated with the Gasification of Walnut Shells for Bioenergy and Biochar Production
Source: PLoS One. 2016 Mar 10;11(3):e0150837. doi: 10.1371/journal.pone.0150837 (PMC4786142; doi:10.1371/journal.pone.0150837)
Supplement: S1 Table — (PDF) [file pone.0150837.s003.pdf]

S1 Table: List of management and precipitation events that occurred at Dixon Ridge Farm (Winters, CA, USA) during the 29 months of this study.

Event	Sampling period	Activity	Date
Event 1	GS1	Irrigation	July 8, 2010
Event 2	GS1	Tillage	July 27, 2010
Event 3	GS1	Irrigation	August 12, 2010
Event 4	GS1	Harvest	October 2, 2010
Event 5	TD1	Precipitation	October 25, 2010
Event 6	TD1	Precipitation	November 8, 2010
Event 7	TD1	Precipitation	November 28, 2010
Event 8	TD1	Mowing	April 8, 2011
Event 9	GS2	Mowing and Irrigation	June 21, 2011
Event 10	GS2	Irrigation	July 26, 2011
Event 11	GS2	Irrigation	August 11, 2011
Event 12	GS2	Fertilization and Irrigation	August 30, 2011
Event 13	GS2	Precipitation and mowing	September 26, 2011
Event 14	GS2	Precipitation	October 6, 2011
Event 15	GS2	Harvest	October 24, 2011
Event 16	TD2	Precipitation	November 4, 2011
Event 17	TD2	Precipitation	January 21, 2012
Event 18	TD2	Precipitation	March 19, 2012
Event 19	TD2	Irrigation	May 27, 2012
Event 20	GS3	Mowing	June 8, 2012
Event 21	GS3	Irrigation	June 16, 2012
Event 22	GS3	Irrigation	June 25, 2012
Event 23	GS3	Harvest	October 22, 2012
