# Supplementary material for: Carbon Abatement and Emissions Associated with the Gasification of Walnut Shells for Bioenergy and Biochar Production
Source: PLoS One. 2016 Mar 10;11(3):e0150837. doi: 10.1371/journal.pone.0150837 (PMC4786142; doi:10.1371/journal.pone.0150837)
Supplement: S2 Table — Shown in parentheses is ± one standard error (n = 3). None of the treatments significantly altered the cumulative CO2–C emissions at p < 0.05. (PDF) [file pone.0150837.s004.pdf]

S2 Table: Cumulative CO_2–C emissions by sampling season from both tree and tractor rows of a walnut orchard in Winters, CA, USA. Shown in parentheses is ± one standard error (n = 3). None of the treatments significantly altered the cumulative CO_2–C emissions at $p < 0.05$.

Row	Treatment	Growing season 1 (2010)	Tree dormancy 1 (2010-2011)	Growing season 2 (2011)	Tree dormancy 2 (2011-2012)	Growing season 3 (2012)
		Mg CO_2-C ha^{-1}				
Tree	Control	2.45 (0.14)	4.20 (1.04)	4.15 (0.25)	2.60 (0.34)	1.52 (0.07)
	Biochar	2.63 (0.09)	3.22 (0.55)	3.53 (0.43)	2.78 (0.28)	1.35 (0.11)
	Compost	2.65 (0.06)	2.96 (0.33)	3.15 (0.38)	3.64 (0.87)	2.10 (0.31)
	Biochar+compost	2.66 (0.20)	2.49 (0.22)	3.21 (0.53)	3.23 (0.58)	1.82 (0.10)
	p-value	*0.66*	*0.18*	*0.35*	*0.59*	*0.06*
		Mg CO_2-C ha^{-1}				
Tractor	Control	2.71 (0.27)	2.85 (0.44)	4.30 (0.83)	5.03 (0.78)	2.22 (0.35)
	Biochar	2.52 (0.25)	3.01 (0.09)	3.89 (0.23)	4.30 (0.31)	2.01 (0.08)
	Compost	2.96 (0.52)	3.22 (0.11)	5.25 (0.81)	4.44 (0.94)	1.78 (0.20)
	Biochar+compost	2.65 (0.26)	2.91 (0.32)	5.53 (0.87)	4.57 (0.62)	2.19 (0.07)
	p-value	*0.82*	*0.81*	*0.4*	*0.89*	*0.47*
