# Supplementary material for: Carbon Abatement and Emissions Associated with the Gasification of Walnut Shells for Bioenergy and Biochar Production
Source: PLoS One. 2016 Mar 10;11(3):e0150837. doi: 10.1371/journal.pone.0150837 (PMC4786142; doi:10.1371/journal.pone.0150837)
Supplement: S3 Table — Shown in parentheses is ± one standard error (n = 3). None of the treatments significantly altered the cumulative N2O-N emissions at p < 0.05. (PDF) [file pone.0150837.s005.pdf]

S3 Table: Cumulative N_2O-N emissions by sampling season from both tree and tractor rows of a walnut orchard in Winters, CA, USA. Shown in parentheses is ± one standard error (n = 3). None of the treatments significantly altered the cumulative N_2O-N emissions at $p < 0.05$.

Row	Treatment	Growing season 1 (2010)	Tree dormancy 1 (2010-2011)	Growing season 2 (2011)	Tree dormancy 2 (2011-2012)	Growing season 3 (2012)
				kg N_2O-N ha^{-1}		
Tree	Control	0.61 (0.15)	0.54 (0.10)	0.88 (0.03)	0.30 (0.05)	0.51 (0.05)
	Biochar	0.48 (0.03)	0.46 (0.18)	1.12 (0.16)	0.21 (0.03)	0.41 (0.16)
	Compost	0.65 (0.01)	0.32 (0.11)	0.79 (0.06)	0.19 (0.02)	0.46 (0.01)
	Biochar+compost	0.78 (0.19)	0.24 (0.05)	1.08 (0.20)	0.18 (0.03)	0.54 (0.14)
	p-value	*0.43*	*0.35*	*0.32*	*0.09*	*0.5*
				kg N_2O-N ha^{-1}		
Tractor	Control	0.93 (0.16)	0.36 (0.03)	1.94 (0.28)	0.47 (0.10)	0.79 (0.08)
	Biochar	0.72 (0.09)	0.30 (0.08)	1.41 (0.10)	0.69 (0.39)	0.62 (0.09)
	Compost	0.95 (0.09)	0.39 (0.09)	2.44 (0.44)	0.65 (0.13)	0.87 (0.27)
	Biochar+compost	0.76 (0.07)	0.55 (0.06)	2.52 (0.66)	0.41 (0.05)	0.83 (0.18)
	p-value	*0.38*	*0.14*	*0.29*	*0.76*	*0.75*
