# Supplementary material for: Carbon Abatement and Emissions Associated with the Gasification of Walnut Shells for Bioenergy and Biochar Production
Source: PLoS One. 2016 Mar 10;11(3):e0150837. doi: 10.1371/journal.pone.0150837 (PMC4786142; doi:10.1371/journal.pone.0150837)
Supplement: S4 Table — Shown in parentheses is ± one standard error (n = 3). None of the treatments significantly altered the cumulative CO2 emissions at p < 0.05. (PDF) [file pone.0150837.s006.pdf]

S4 Table: Cumulative CO_2 emissions by event that occurred during growing season 1 (GS1), period between June and October 2010, from both tree and tractor rows of a walnut orchard in Winters, CA, USA. Shown in parentheses is ± one standard error (n = 3). None of the treatments significantly altered the cumulative CO_2 emissions at $p < 0.05$.

Location	Treatment	Event 1 *Irrigation*	Event 2 *Tillage*	Event 3 *Irrigation*	Event 4 *Harvest*
		Mg CO_2-C ha^{-1}			
Tree row	Control	0.43 (0.02)	0.43 (0.05)	0.21 (0.06)	0.36 (0.06)
	Biochar	0.47 (0.05)	0.52 (0.17)	0.32 (0.10)	0.30 (0.06)
	Compost	0.57 (0.08)	0.22 (0.02)	0.24 (0.02)	0.46 (0.06)
	Biochar+compost	0.45 (0.03)	0.48 (0.07)	0.32 (0.01)	0.45 (0.08)
	p-value	*0.28*	*0.22*	*0.45*	*0.31*
		Mg CO_2-C ha^{-1}			
Tractor row	Control	0.54 (0.10)	0.33 (0.05)	0.34 (0.01)	0.31 (0.05)
	Biochar	0.59 (0.06)	0.33 (0.09)	0.23 (0.05)	0.21 (0.06)
	Compost	0.65 (0.19)	0.36 (0.06)	0.35 (0.07)	0.29 (0.01)
	Biochar+compost	0.55 (0.01)	0.42 (0.08)	0.31 (0.07)	0.25 (0.07)
	p-value	*0.91*	*0.81*	*0.48*	*0.55*
