# Supplementary material for: Carbon Abatement and Emissions Associated with the Gasification of Walnut Shells for Bioenergy and Biochar Production
Source: PLoS One. 2016 Mar 10;11(3):e0150837. doi: 10.1371/journal.pone.0150837 (PMC4786142; doi:10.1371/journal.pone.0150837)
Supplement: S5 Table — Shown in parentheses is ± one standard error (n = 3). None of the treatments significantly altered the cumulative CO2 emissions at p < 0.05. (PDF) [file pone.0150837.s007.pdf]

S5 Table: Cumulative CO_2 emissions by event that occurred during tree dormancy 1 (TD1), period between November 2010 and May 2011, from both tree and tractor rows of a walnut orchard in Winters, CA, USA. Shown in parentheses is ± one standard error (n = 3). None of the treatments significantly altered the cumulative CO_2 emissions at $p < 0.05$.

Location	Treatment	Event 5 *Precipitation*	Event 6 *Precipitation*	Event 7 *Precipitation*	Event 8 *Mowing*
		Mg CO_2-C ha^{-1}			
Tree row	Control	0.23 (0.04)	0.21 (0.01)	0.19 (0.01)	0.52 (0.03)
	Biochar	0.17 (0.07)	0.20 (0.02)	0.16 (0.05)	0.51 (0.12)
	Compost	0.20 (0.03)	0.21 (0.04)	0.14 (0.03)	0.33 (0.09)
	Biochar+compost	0.16 (0.02)	0.24 (0.10)	0.12 (0.03)	0.35 (0.03)
	p-value	*0.70*	*0.94*	*0.49*	*0.25*
		Mg CO_2-C ha^{-1}			
Tractor row	Control	0.13 (0.03)	0.16 (0.03)	0.10 (0.01) b	0.46 (0.06)
	Biochar	0.14 (0.00)	0.14 (0.01)	0.16 (0.01) b	0.31 (0.06)
	Compost	0.17 (0.03)	0.21 (0.03)	0.18 (0.02) a	0.45 (0.05)
	Biochar+compost	0.14 (0.04)	0.23 (0.05)	0.14 (0.02) ab	0.35 (0.05)
	p-value	*0.73*	*0.21*	*0.03*	*0.21*
