# Supplementary material for: Carbon Abatement and Emissions Associated with the Gasification of Walnut Shells for Bioenergy and Biochar Production
Source: PLoS One. 2016 Mar 10;11(3):e0150837. doi: 10.1371/journal.pone.0150837 (PMC4786142; doi:10.1371/journal.pone.0150837)
Supplement: S6 Table — Shown in parentheses is ± one standard error (n = 3). None of the treatments significantly altered the cumulative CO2 emissions at p < 0.05. (PDF) [file pone.0150837.s008.pdf]

**S6 Table**: Cumulative $CO_2$ emissions by event that occurred during growing season 2 (GS2), period between June and October 2011, from both tree and tractor rows of a walnut orchard in Winters, CA, USA. Shown in parentheses is ± one standard error (n = 3). None of the treatments significantly altered the cumulative $CO_2$ emissions at $p < 0.05$.

| Location | Treatment | Event 9 *Irrig+Mow* | Event 10 *Irrigation* | Event 11 *Irrigation* | Event 12 *Fertil+Irrig* | Event 13 *Mow+Precip* | Event 14 *Precip* | Event 15 *Harvest* |
|---|---|---|---|---|---|---|---|---|
| | | | | | Mg $CO_2$-C $ha^{-1}$ | | | |
| Tree row | Control | 0.45 (0.04) | 0.29 (0.07)ab | 0.27 (0.03) | 0.48 (0.04) | 0.26 (0.02) | 0.29 (0.02)ab | 0.13 (0.01) |
| | Biochar | 0.25 (0.01) | 0.14 (0.01)b | 0.22 (0.07) | 0.44 (0.03) | 0.28 (0.03) | 0.39 (0.02)a | 0.09 (0.00) |
| | Compost | 0.43 (0.10) | 0.24 (0.02)ab | 0.28 (0.08) | 0.40 (0.07) | 0.21 (0.10) | 0.24 (0.07)b | 0.10 (0.02) |
| | Biochar+compost | 0.38 (0.04) | 0.33 (0.02) a | 0.32 (0.13) | 0.33 (0.07) | 0.22 (0.07) | 0.28 (0.00)ab | 0.09 (0.02) |
| | *p-value* | *0.15* | *0.06* | *0.88* | *0.28* | *0.79* | *0.10* | *0.38* |
| | | | | | Mg $CO_2$-C $ha^{-1}$ | | | |
| Tractor row | Control | 0.50 (0.10) b | 0.30 (0.01) | 0.39 (0.16) | 0.65 (0.12) | 0.24 (0.01) | 0.49 (0.02) | 0.23 (0.03) a |
| | Biochar | 0.49 (0.04) b | 0.33 (0.06) | 0.48 (0.10) | 0.70 (0.16) | 0.31 (0.09) | 0.50 (0.05) | 0.17 (0.02)ab |
| | Compost | 0.88 (0.12) a | 0.35 (0.08) | 0.56 (0.24) | 0.85 (0.21) | 0.31 (0.01) | 0.58 (0.05) | 0.11 (0.01) b |
| | Biochar+compost | 0.74 (0.13) ab | 0.51 (0.10) | 0.52 (0.07) | 0.52 (0.05) | 0.25 (0.03) | 0.49 (0.10) | 0.15 (0.03)ab |
| | *p-value* | *0.07* | *0.24* | *0.88* | *0.49* | *0.63* | *0.68* | *0.06* |
