# Supplementary material for: Carbon Abatement and Emissions Associated with the Gasification of Walnut Shells for Bioenergy and Biochar Production
Source: PLoS One. 2016 Mar 10;11(3):e0150837. doi: 10.1371/journal.pone.0150837 (PMC4786142; doi:10.1371/journal.pone.0150837)
Supplement: S7 Table — Shown in parentheses is ± one standard error (n = 3). None of the treatments significantly altered the cumulative CO2 emissions at p < 0.05. (PDF) [file pone.0150837.s009.pdf]

S7 Table: Cumulative CO_2 emissions by event that occurred during tree dormancy 2 (TD2), period between November 2011 and May 2012, from both tree and tractor rows of a walnut orchard in Winters, CA, USA. Shown in parentheses is ± one standard error (n = 3). None of the treatments significantly altered the cumulative CO_2 emissions at $p < 0.05$.

Location	Treatment	Event 16 *Precipitation*	Event 17 *Precipitation*	Event 18 *Precipitation*	Event 19 *Irrigation*
		Mg CO_2-C ha^{-1}			
Tree row	Control	0.19 (0.04)	0.43 (0.08)	0.22 (0.09)	0.13 (0.02)
	Biochar	0.18 (0.04)	0.51 (0.10)	0.28 (0.03)	0.15 (0.01)
	Compost	0.16 (0.02)	0.54 (0.12)	0.45 (0.21)	0.17 (0.01)
	Biochar+compost	0.23 (0.02)	0.55 (0.12)	0.25 (0.03)	0.16 (0.01)
	p-value	*0.54*	*0.85*	*0.53*	*0.32*
		Mg CO_2-C ha^{-1}			
Tractor row	Control	0.52 (0.11)	0.71 (0.08)	0.49 (0.06)	0.31 (0.07)
	Biochar	0.42 (0.05)	0.61 (0.09)	0.44 (0.04)	0.23 (0.03)
	Compost	0.33 (0.03)	0.85 (0.44)	0.54 (0.04)	0.19 (0.04)
	Biochar+compost	0.47 (0.05)	0.88 (0.44)	0.36 (0.13)	0.26 (0.02)
	p-value	*0.32*	*0.83*	*0.42*	*0.30*
