# Supplementary material for: Carbon Abatement and Emissions Associated with the Gasification of Walnut Shells for Bioenergy and Biochar Production
Source: PLoS One. 2016 Mar 10;11(3):e0150837. doi: 10.1371/journal.pone.0150837 (PMC4786142; doi:10.1371/journal.pone.0150837)
Supplement: S8 Table — Shown in parentheses is ± one standard error (n = 3). Means followed by different letter within a column are statistically different at p > 0.05. (PDF) [file pone.0150837.s010.pdf]

S8 Table: Cumulative CO_2 emissions by event that occurred during growing season 3 (GS3), period between June and October 2012, from both tree and tractor rows of a walnut orchard in Winters, CA, USA. Shown in parentheses is ± one standard error (n = 3). Means followed by different letter within a column are statistically different at *p* > 0.05.

Location	Treatment	Event 20 *Mowing*	Event 21 *Irrigation*	Event 22 *Irrigation*	Event 23 *Harvest*
		Mg CO_2-C ha^{-1}			
Tree row	Control	0.06 (0.01)	0.10 (0.02)	0.13 (0.04)	0.06 (0.01)
	Biochar	0.07 (0.00)	0.09 (0.01)	0.17 (0.04)	0.03 (0.01)
	Compost	0.11 (0.02)	0.13 (0.02)	0.13 (0.01)	0.06 (0.01)
	Biochar+compost	0.09 (0.02)	0.09 (0.02)	0.20 (0.03)	0.05 (0.01)
	p-value	*0.21*	*0.29*	*0.38*	*0.30*
		Mg CO_2-C ha^{-1}			
Tractor row	Control	0.13 (0.03)	0.12 (0.01) bc	0.17 (0.04)	0.09 (0.01) a
	Biochar	0.10 (0.01)	0.14 (0.01) ab	0.19 (0.02)	0.04 (0.01) b
	Compost	0.11 (0.01)	0.11 (0.01) c	0.16 (0.03)	0.07 (0.01) ab
	Biochar+compost	0.12 (0.01)	0.17 (0.01) a	0.17 (0.01)	0.05 (0.01) ab
	p-value	*0.73*	*0.00*	*0.86*	*0.06*
