# Supplementary material for: Carbon Abatement and Emissions Associated with the Gasification of Walnut Shells for Bioenergy and Biochar Production
Source: PLoS One. 2016 Mar 10;11(3):e0150837. doi: 10.1371/journal.pone.0150837 (PMC4786142; doi:10.1371/journal.pone.0150837)
Supplement: S9 Table — Shown in parentheses is ± one standard error (n = 3). None of the treatments significantly altered the cumulative N2O emissions at p < 0.05. (PDF) [file pone.0150837.s011.pdf]

S9 Table: Cumulative N_2O emissions by event that occurred during growing season 1 (GS1), period between June and October 2010, from both tree and tractor rows of a walnut orchard in Winters, CA, USA. Shown in parentheses is ± one standard error (n = 3). None of the treatments significantly altered the cumulative N_2O emissions at $p < 0.05$.

Location	Treatment	Event 1 *Irrigation*	Event 2 *Tillage*	Event 3 *Irrigation*	Event 4 *Harvest*
		kg N_2O-N ha^{-1}			
Tree row	Control	0.13 (0.07)	0.08 (0.02) ab	0.05 (0.01)	0.05 (0.01)
	Biochar	0.06 (0.02)	0.10 (0.02) ab	0.05 (0.01)	0.05 (0.01)
	Compost	0.11 (0.03)	0.04 (0.01) b	0.04 (0.01)	0.05 (0.00)
	Biochar+compost	0.16 (0.07)	0.11 (0.02) a	0.07 (0.02)	0.08 (0.02)
	p-value	*0.33*	*0.09*	*0.67*	*0.56*
		kg N_2O-N ha^{-1}			
Tractor row	Control	0.15 (0.03)	0.18 (0.02)	0.10 (0.01)	0.12 (0.04)
	Biochar	0.09 (0.02)	0.12(0.03)	0.06 (0.02)	0.09 (0.02)
	Compost	0.17 (0.06)	0.11 (0.02)	0.10 (0.03)	0.18 (0.03)
	Biochar+compost	0.12 (0.03)	0.12 (0.02)	0.08 (0.01)	0.06 (0.02)
	p-value	*0.54*	*0.45*	*0.33*	*0.11*
