# Supplementary material for: Carbon Abatement and Emissions Associated with the Gasification of Walnut Shells for Bioenergy and Biochar Production
Source: PLoS One. 2016 Mar 10;11(3):e0150837. doi: 10.1371/journal.pone.0150837 (PMC4786142; doi:10.1371/journal.pone.0150837)
Supplement: S10 Table — Shown in parentheses is ± one standard error (n = 3). Means followed by different letter within a column are statistically different at p > 0.05. (PDF) [file pone.0150837.s012.pdf]

S10 Table: Cumulative N_2O emissions by event that occurred during tree dormancy 1 (TD1), period between November 2010 and May 2011, from both tree and tractor rows of a walnut orchard in Winters, CA, USA. Shown in parentheses is ± one standard error (n = 3). Means followed by different letter within a column are statistically different at *p* > 0.05.

Location	Treatment	Event 5 *Precipitation*	Event 6 *Precipitation*	Event 7 *Precipitation*	Event 8 *Mowing*
			kg N_2O-N ha^{-1}		
Tree row	Control	0.03 (0.01) a	0.04 (0.02)	0.02 (0.01)	0.07 (0.04)
	Biochar	0.02 (0.00) b	0.03 (0.00)	0.01 (0.00)	0.08 (0.03)
	Compost	0.01 (0.00) ab	0.02 (0.00)	0.02 (0.00)	0.04 (0.02)
	Biochar+compost	0.03 (0.01) ab	0.02 (0.01)	0.01 (0.00)	0.03 (0.00)
	p-value	*0.06*	*0.67*	*0.29*	*0.66*
			kg N_2O-N ha^{-1}		
Tractor row	Control	0.03 (0.01)	0.02 (0.01) ab	0.01 (0.00)	0.05 (0.03)
	Biochar	0.07 (0.05)	0.01 (0.00) b	0.01 (0.00)	0.02 (0.01)
	Compost	0.05 (0.02)	0.03 (0.01) a	0.02 (0.00)	0.02 (0.01)
	Biochar+compost	0.03 (0.01)	0.01 (0.0) b	0.01 (0.01)	0.04 (0.02)
	p-value	*0.97*	*0.03*	*0.61*	*0.87*
