# Supplementary material for: Carbon Abatement and Emissions Associated with the Gasification of Walnut Shells for Bioenergy and Biochar Production
Source: PLoS One. 2016 Mar 10;11(3):e0150837. doi: 10.1371/journal.pone.0150837 (PMC4786142; doi:10.1371/journal.pone.0150837)
Supplement: S11 Table — Shown in parentheses is ± one standard error (n = 3). Means followed by different letter within a column are statistically different at p > 0.05. (PDF) [file pone.0150837.s013.pdf]

S11 Table: Cumulative N_2O emissions by event that occurred during growing season 2 (GS2), period between June and October 2011, from both tree and tractor rows of a walnut orchard in Winters, CA, USA. Shown in parentheses is ± one standard error (n = 3). Means followed by different letter within a column are statistically different at $p > 0.05$.

Location	Treatment	Event 9 *Irrig+Mow*	Event 10 *Irrigation*	Event 11 *Irrigation*	Event 12 *Fertil+Irrig*	Event 13 *Mow+Precip*	Event 14 *Precip*	Event 15 *Harvest*
					kg N_2O-N ha^{-1}			
Tree row	Control	0.12 (0.05)	0.07 (0.02) a	0.04 (0.01)	0.11 (0.02)	0.18 (0.04)	0.13 (0.03)	0.01 (0.00)
	Biochar	0.09 (0.03)	0.02 (0.00) b	0.02 (0.01)	0.27 (0.04)	0.41 (0.07)	0.21 (0.04)	0.01 (0.00)
	Compost	0.13 (0.03)	0.05 (0.01)ab	0.02 (0.00)	0.16 (0.05)	0.19 (0.04)	0.11 (0.03)	0.01 (0.00)
	Biochar+compost	0.07 (0.01)	0.05 (0.01) ab	0.03 (0.01)	0.11 (0.02)	0.48 (0.24)	0.16 (0.02)	0.02 (0.01)
	p-value	*0.42*	*0.02*	*0.25*	*0.13*	*0.24*	*0.27*	*0.56*
					kg N_2O-N ha^{-1}			
Tractor row	Control	0.16 (0.05)	0.05 (0.00)	0.18 (0.16)	0.46 (0.17)	0.48 (0.06)	0.41 (0.05) ab	0.06 (0.03)
	Biochar	0.23 (0.08)	0.04 (0.00)	0.04 (0.02)	0.33 (0.06)	0.27 (0.09)	0.29 (0.08) b	0.05 (0.02)
	Compost	0.38 (0.18)	0.06 (0.03)	0.07 (0.04)	0.41 (0.11)	0.53 (0.11)	0.76 (0.06) a	0.02 (0.01)
	Biochar+compost	0.17 (0.03)	0.03 (0.01)	0.04 (0.02)	0.20 (0.11)	1.16 (0.58)	0.63 (0.20) ab	0.01 (0.01)
	p-value	*0.49*	*0.39*	*0.78*	*0.28*	*0.26*	*0.05*	*0.26*
