# Supplementary material for: Carbon Abatement and Emissions Associated with the Gasification of Walnut Shells for Bioenergy and Biochar Production
Source: PLoS One. 2016 Mar 10;11(3):e0150837. doi: 10.1371/journal.pone.0150837 (PMC4786142; doi:10.1371/journal.pone.0150837)
Supplement: S12 Table — Shown in parentheses is ± one standard error (n = 3). None of the treatments significantly altered the cumulative N2O emissions at p < 0.05. (PDF) [file pone.0150837.s014.pdf]

S12 Table: Cumulative N_2O emissions by event that occurred during tree dormancy 1 (TD1), period between November 2010 and May 2011, from both tree and tractor rows of a walnut orchard in Winters, CA, USA. Shown in parentheses is ± one standard error (n = 3). None of the treatments significantly altered the cumulative N_2O emissions at $p < 0.05$.

Location	Treatments	Event 16 *Precipitation*	Event 17 *Precipitation*	Event 18 *Precipitation*	Event 19 *Irrigation*
		kg N_2O-N ha^{-1}			
Tree row	Control	0.05 (0.02)	0.05 (0.03)	0.01 (0.01)	0.03 (0.01)
	Biochar	0.07 (0.02)	0.04 (0.01)	0.01 (0.01)	0.01 (0.01)
	Compost	0.03 (0.00)	0.03 (0.01)	0.01 (0.01)	0.03 (0.01)
	Biochar+compost	0.06 (0.03)	0.03 (0.01)	0.00 (0.00)	0.03 (0.01)
	p-value	*0.57*	*0.96*	*0.68*	*0.24*
		kg N_2O-N ha^{-1}			
Tractor row	Control	0.18 (0.03)	0.04 (0.00)	0.01 (0.01)	0.03 (0.01)
	Biochar	0.38 (0.28)	0.06 (0.01)	0.01 (0.00)	0.03 (0.01)
	Compost	0.29 (0.09)	0.06 (0.03)	0.01 (0.01)	0.02 (0.01)
	Biochar+compost	0.13 (0.05)	0.07 (0.03)	0.00 (0.00)	0.07 (0.03)
	p-value	*0.65*	*0.80*	*0.88*	*0.44*
