# Supplementary material for: Carbon Abatement and Emissions Associated with the Gasification of Walnut Shells for Bioenergy and Biochar Production
Source: PLoS One. 2016 Mar 10;11(3):e0150837. doi: 10.1371/journal.pone.0150837 (PMC4786142; doi:10.1371/journal.pone.0150837)
Supplement: S13 Table — Shown in parentheses is ± one standard error (n = 3). None of the treatments significantly altered the cumulative N2O emissions at p < 0.05. (PDF) [file pone.0150837.s015.pdf]

S13 Table: Cumulative N_2O emissions by event that occurred during growing season 1 (GS1), period between June and October 2010, from both tree and tractor rows of a walnut orchard in Winters, CA, USA. Shown in parentheses is ± one standard error (n = 3). None of the treatments significantly altered the cumulative N_2O emissions at $p < 0.05$.

Location	Treatments	Event 20 *Mowing*	Event 21 *Irrigation*	Event 22 *Irrigation*	Event 23 *Harvest*
		kg N_2O-N ha^{-1}			
Tree row	Control	0.03 (0.00)	0.03 (0.00)	0.05 (0.01)	0.03 (0.01)
	Biochar	0.02 (0.00)	0.02 (0.01)	0.04 (0.01)	0.03 (0.03)
	Compost	0.04 (0.00)	0.04 (0.00)	0.04 (0.01)	0.02 (0.00)
	Biochar+compost	0.04 (0.03)	0.04 (0.01)	0.07 (0.01)	0.03 (0.01)
	p-value	*0.55*	*0.44*	*0.35*	*0.68*
		kg N_2O-N ha^{-1}			
Tractor row	Control	0.03 (0.02)	0.03 (0.00)	0.09 (0.04)	0.09 (0.04)
	Biochar	0.07 (0.04)	0.05 (0.01)	0.10 (0.04)	0.02 (0.00)
	Compost	0.02 (0.01)	0.03 (0.01)	0.08 (0.03)	0.09 (0.05)
	Biochar+compost	0.12 (0.05)	0.07 (0.03)	0.07 (0.02)	0.08 (0.02)
	p-value	*0.41*	*0.53*	*0.95*	*0.17*
